# Microsatellite-based genetic diversity assessment of Donggala cattle *(Bos indicus)* in Indonesia: Insights for conservation and breeding

**DOI:** 10.14202/vetworld.2025.2981-2990

**Published:** 2025-10-08

**Authors:** Yulius Duma, Paskah Partogi Agung, Mobius Tanari, Amirudin Dg. Malewa, Muhammad Ilyas Mumu, Syahruddin Said, Ferdy Saputra, Ekayanti Mulyawati Kaiin, Muhammad Gunawan, Tulus Maulana, Nina Herlina, Damry Damry

**Affiliations:** 1 Animal Science, Faculty of Animal Husbandry and Fisheries, Tadulako University, Jl. Soekarno Hatta KM. 9, Palu 94117, Central Sulawesi, Indonesia; 2 Research Center for Applied Zoology, National Research and Innovation Agency-BRIN, Jl. Raya Bogor KM. 46, Cibinong 16911, West Java, Indonesia; 3 Research Center for Animal Husbandry, National Research and Innovation Agency-BRIN, Jl. Raya Bogor KM. 46, Cibinong 16911, West Java, Indonesia

**Keywords:** 3D principal component analyses, breeding, conservation, Donggala cattle, genetic diversity, microsatellite markers

## Abstract

**Background and Aim:**

Donggala cattle (*Bos indicus*), indigenous to Central Sulawesi, Indonesia, are recognized for their productive and reproductive performance. However, molecular information on their genetic diversity is scarce. Understanding genetic variability is essential for sustainable conservation and targeted breeding strategies. This study aimed to characterize the genetic diversity and relationships of Donggala cattle using microsatellite markers and advanced multivariate analyses.

**Materials and Methods:**

Seventy-five blood samples were collected from unrelated Donggala cattle in Central Sulawesi. Genomic DNA was extracted and amplified across ten Food and Agriculture Organization-recommended microsatellite loci. Allele frequency, observed heterozygosity (Ho), expected heterozygosity, and polymorphism information content (PIC) were calculated. Genetic distances and clustering were assessed using Unweighted Pair Group Method with Arithmetic Mean (UPGMA) and analysis of molecular variance (AMOVA). Two- and three-dimensional principal component analysis (PCA) was conducted to visualize genetic differentiation, with comparative datasets from other Indonesian cattle breeds.

**Results:**

High allelic diversity was detected, with SPS113 (12 alleles), ETH225 (11 alleles), and TGLA122 (9 alleles) being the most informative markers (PIC: 0.80–0.84). Ho was highest at ETH225 (0.95), reflecting substantial genetic variation. UPGMA and admixture analyses placed Donggala cattle closest to Pesisir cattle, though phenotypically more similar to larger *B. indicus* breeds such as Ongole Grade. The 3D PCA provided enhanced discriminatory power, distinctly separating Donggala from exotic and crossbred cattle and differentiating Bali cattle from Banteng. AMOVA indicated that 22% of genetic variation existed among populations, while 21% was among individuals.

**Conclusion:**

Donggala cattle exhibit considerable genetic diversity, underscoring their value as a reservoir for breeding and conservation programs. Microsatellite markers, particularly SPS113, ETH225, and TGLA122, proved highly informative for genetic assessment. The application of 3D PCA enhanced resolution in distinguishing closely related breeds, supporting its use in molecular characterization. These findings provide essential baseline data for sustainable management, conservation, and genetic improvement of Donggala cattle.

## INTRODUCTION

Donggala cattle (*Bos indicus*) is one of Indonesia’s recognized indigenous breeds, formally established under the Decree of the Minister of Agriculture of the Republic of Indonesia No. 666/Kpts/SR.120/6/2014. Originating from Donggala Regency in Central Sulawesi Province, these cattle are primarily raised for beef production and play a vital role in the region’s agricultural and socioeconomic systems. On average, mature Donggala bulls weigh about 317 kg, while adult cows reach approximately 195 kg [1].

Despite their significance, limited research has addressed the genetic diversity of Donggala cattle. Comprehensive insights into their genetic variability are crucial for designing effective conservation strategies, particularly for safeguarding Indonesian indigenous breeds. Such knowledge also underpins the development of sustainable breeding enterprises that can benefit local farmers. Advances in molecular genetics now enable detailed DNA-level investigations, with microsatellite markers emerging as a valuable tool for pedigree analysis and assessing genetic relationships among populations [2]. These markers are highly polymorphic, codominant, and widely distributed across the genome [3, 4], making them especially useful for evaluating genetic diversity and genetic distances [5]. The Food and Agriculture Organization (FAO) of the United Nations has recommended a standard panel of 30 microsatellite markers for assessing genetic variation and relationships among cattle breeds [6–9]. Within Indonesia, 12 of these markers have already been successfully applied in studies of *B. indicus*, *Bos taurus*, and *Bos javanicus* populations [10].

Although Donggala cattle have been officially recognized as an indigenous Indonesian breed, scientific information on their genetic makeup remains extremely limited. Most molecular characterization studies in Indonesia have focused on other breeds such as Bali, Pesisir, Ongole Grade, and Madura, leaving Donggala cattle largely underexplored. Without detailed genetic data, the conservation status, population structure, and breeding potential of Donggala cattle cannot be accurately assessed. Furthermore, while microsatellite DNA markers have been widely applied to evaluate genetic diversity in various *B. indicu*s, *B. tauru*s, and *B. javanicu*s populations, no published studies have yet applied these molecular tools to Donggala cattle. This lack of molecular-level information creates a critical knowledge gap that restricts the formulation of evidence-based conservation and sustainable breeding programs for this indigenous resource.

The present study aimed to assess the genetic diversity and population structure of Donggala cattle using FAO-recommended microsatellite markers. Specifically, the objectives were to (i) evaluate allelic richness, heterozygosity, and polymorphism information content (PIC) across key loci, (ii) determine genetic distances and relationships between Donggala and other Indonesian cattle breeds, and (iii) apply advanced multivariate approaches, including 3D principal component analysis (PCA), to visualize genetic clustering and breed differentiation. By filling this knowledge gap, the study seeks to provide foundational molecular data that can support conservation efforts, strengthen genetic improvement programs, and enhance the long-term sustainability of Donggala cattle.

## MATERIALS AND METHODS

### Ethical approval

All experimental procedures involving animals were approved by the Ethical Clearance Committee of the National Research and Innovation Agency (BRIN), Jakarta, Indonesia (Approval No. 077/KE.02/SK/10/2022). All protocols were conducted in accordance with relevant animal welfare regulations.

### Study period and location

The study was conducted from May to July 2023 in the sub-districts of Banawa Selatan and Banawa Tengah, Central Sulawesi Province.

### Sampling sites and blood collection

A total of 75 peripheral blood samples were collected from healthy, unrelated Donggala cattle. Blood was drawn from the coccygeal vein using sterile 18G needles and stored in ethylenediaminetetraacetic acid-coated Vacutainer tubes (Vaculab, China). Samples were maintained at 4°C during transport and processed within 24 h for DNA extraction.

### DNA extraction and quality assessment

Genomic DNA was extracted using the DNeasy Blood and Tissue Kit (Qiagen, Germany) following the manufacturer’s protocol. DNA quality and concentration were assessed using a NanoDrop spectrophotometer (Thermo Fisher Scientific, USA), and integrity was verified by 1.5% agarose gel electrophoresis.

### Microsatellite marker selection and PCR amplification

Ten microsatellite loci from the 30 recommended by FAO [11] were selected based on previous validation in *B. indicus* breeds and their high polymorphic potential. Primer sequences, annealing temperatures, expected amplicon sizes, and labeling details followed the protocol of Agung *et al*. [12]. PCR reactions (32.2 μL) contained 18 μL of KAPA2G Robust HotStart ReadyMix (1^st^ BASE, Malaysia), 2.8 μL of primer mix, 10 μL of nuclease-free water, and 1.4 μL of DNA template. Cycling conditions were as follows: 94°C for 5 min; 35 cycles of 94°C for 30 s, 51–59°C for 30 s, and 72°C for 30 s; final extension at 72°C for 5 min. Both positive and negative controls were included to ensure amplification, reliability and specificity.

### Capillary electrophoresis and genotyping

PCR products were multiplexed and analyzed through capillary electrophoresis using an ABI 3730 Genetic Analyzer at the 1^st^ BASE Laboratory (Malaysia). Allele sizes were determined with GeneMapper software 6 (Thermo Fisher) using internal size standards, and genotyping accuracy was manually verified.

### Genetic diversity analysis

Genetic parameters, including allele number, allele frequency, observed heterozygosity (Ho), expected heterozygosity (He), and PIC, were estimated using Cervus v3.0 [13]. Nei’s genetic distances and Unweighted Pair Group Method with Arithmetic Mean (UPGMA) dendrograms were generated using POPGENE v1.32 [14]. Hardy–Weinberg equilibrium was tested for each locus.

### Population structure and breed comparison

Comparative genotype data from other Indonesian cattle breeds were obtained from Agung *et al*. [12]. Analyses were performed in R v4.5.1 [15] using adegenet [16], ade4 [17], ggplot2, factoextra, plotly, RColorBrewer, htmlwidgets, ggrepel, and dplyr. Genotypes in GENEPOP format were imported using a three-digit allele coding scheme. The optimal number of genetic clusters (K) was determined using find.clusters with the lowest Bayesian information criterion. Discriminant analysis of principal components was conducted using 20 retained principal components and K−1 discriminant axes, with results visualized in admixture bar plots. PCA was performed on scaled genotypes, and both 2D (ggplot2) and interactive 3D (plotly) plots were generated.

### Bottleneck and molecular variance analyses

Population bottleneck tests were conducted using Bottleneck v1.2.02 [18] under the infinite allele model (IAM), two-phase model (TPM), and stepwise mutation model (SMM). Significance was assessed with the Wilcoxon signed-rank test, and allele frequency mode-shift tests were used to detect deviations from mutation-drift equilibrium. Analysis of molecular variance (AMOVA) was performed usingGenAlEx v6.1 [19] to partition genetic variation within and among populations.

## RESULTS

### Allelic diversity and frequency

Microsatellite analysis revealed variation in allelic richness across loci. The SPS113 locus exhibited the highest allelic diversity with 12 alleles, whereas BM1818 showed the lowest with only 3 alleles (Table 1). Allele frequency analysis indicated that allele 264 at BM1818 was the most frequent (0.8421), while allele 181 at BM1824 displayed a frequency of 0.421. These findings suggest that certain alleles dominate specific loci within the Donggala cattle population.

**Table 1:** Frequency of microsatellite alleles in Donggala cattle.

Locus	Allele frequency											
	177	181	183	185	193							
BM1824	0.0263	0.4211	0.3684	0.1579	0.0263							
	281	287	291	293	295	297	299					
ILST6	0.0294	0.0294	0.1471	0.2647	0.2647	0.2059	0.0588					
	117	119	121	123	125	127	129					
TGLA126	0.3684	0.0263	0.1053	0.2105	0.2105	0.0526	0.0263					
	119	131	133	137	139	141	143	145	147	149	157	169
SPS113	0.0263	0.1842	0.0263	0.2895	0.1053	0.1842	0.0263	0.0263	0.0526	0.0263	0.0263	0.0263
	71	77	79	81	83							
TGLA227	0.0286	0.8143	0.0714	0.0571	0.0286							
	136	144	146	150	152	154	162	164	168			
TGLA122	0.1806	0.1111	0.0278	0.1389	0.1389	0.1528	0.1944	0.0139	0.0417			
	135	139	141	143	145	149	151	153	155	157	163	
ETH225	0.1842	0.0263	0.0263	0.2368	0.0263	0.0263	0.0263	0.0789	0.2632	0.0263	0.0789	
	242	244	246	252	254							
SPS115	0.1563	0.5313	0.1250	0.1250	0.0625							
	252	262	264									
BM1818	0.0526	0.1053	0.8421									
	178	180	184	188	192	198	220	284				
CSSM66	0.5294	0.0882	0.0882	0.0294	0.0294	0.1176	0.0882	0.0294				

### PIC and heterozygosity

Among the loci analyzed, SPS113 (12 alleles), ETH225 (11 alleles), and TGLA122 (9 alleles) demonstrated the highest allelic richness. Correspondingly, these loci exhibited the highest PIC, with values of 0.84 for TGLA122, 0.81 for SPS113, and 0.80 for ETH225 (Table 2) [12]. In terms of heterozygosity, the highest observed values were recorded for ETH225 (0.95), TGLA122 (0.83), and SPS113 (0.74), confirming their importance in reflecting the substantial genetic diversity of Donggala cattle.

**Table 2:** Statistical summary of loci in Donggala cattle and local Indonesian cattle breeds.

Loci	Na		Ho		He		PIC	
			
	Donggala	All breed*	Donggala	All breed*	Donggala	All breed*	Donggala	All breed*
BM1824	5	23	0.26	0.45	0.68	0.85	0.60	0.84
ILST6	7	26	0.53	0.45	0.82	0.89	0.76	0.88
TGLA126	7	23	0.26	0.60	0.78	0.93	0.73	0.93
SPS113	12	32	0.74	0.43	0.85	0.89	0.81	0.88
TGLA227	5	31	0.23	0.72	0.33	0.92	0.31	0.92
TGLA122	9	32	0.83	0.75	0.87	0.94	0.84	0.93
ETH225	11	29	0.95	0.88	0.85	0.92	0.80	0.91
SPS115	5	19	0.25	0.53	0.68	0.82	0.62	0.80
BM1818	3	14	0.11	0.38	0.28	0.77	0.26	0.74
CSSM66	8	27	0.71	0.60	0.70	0.76	0.66	0.74
Average			0.49	0.58	0.68	0.87	0.64	0.86

Na = Number of alleles, Ho = Observed heterozygosity, He = Expected heterozygosity, PIC = Polymorphism information content;

* for this analysis, data obtained from Agung *et al*. [12] were merged with additional samples generated in the present study

### Bottleneck and molecular variance analysis

Bottleneck analysis using the Wilcoxon signed-rank test under three mutation models (IAM, TPM, and SMM) revealed significant heterozygosity excess (p < 0.05) in four breeds: Simmental Crossbred, Bali, Madura, and Pasundan (Table 3) [12]. In contrast, Donggala cattle did not show significant bottleneck signals. AMOVA demonstrated that variation among populations accounted for 22% of the total variance, while variation among individuals within populations contributed 21% (Table 4).

**Table 3:** Probability values for bottleneck analysis in a Wilcoxon signed-rank test in 11 native Indonesian cattle populations in three mutation models.

Breed	The infinite allele model	Two-phase model	The stepwise mutation model
Simmental purebred*	0.08	0.65	0.95
Simmental crossbred*	0.01	0.34	0.88
Ongole grade*	0.09	0.61	0.98
Bali*	0.02	0.12	0.37
Pesisir*	0.15	0.54	0.97
Holstein Friesian*	0.24	0.71	0.98
Sumba Ongole*	0.46	0.98	0.99
Madura*	0.05	0.21	0.91
Banteng*	0.57	0.88	0.98
Pasundan*	0.04	0.16	0.99
Donggala	0.31	0.81	0.99

* For the purpose of this analysis, data obtained from Agung *et al*. [12] were merged with additional samples generated in this study

**Table 4:** Analysis of molecular variance among the 11 cattle breeds.

Source	df	SS	MS	Est. Var.	Percentage
Among population	10	533.540	53.354	1.017	22
Among individual	256	1,140.929	4.457	0.948	21
Within individual	267	683.500	2.560	2.560	57
Total	533	2,357.968		4.525	100

df = Degrees of freedom; SS = Sum of squared, MS = Mean of squared, Est. Var. = Estimated variance

### Genetic relationship and clustering

**Figure 1:**
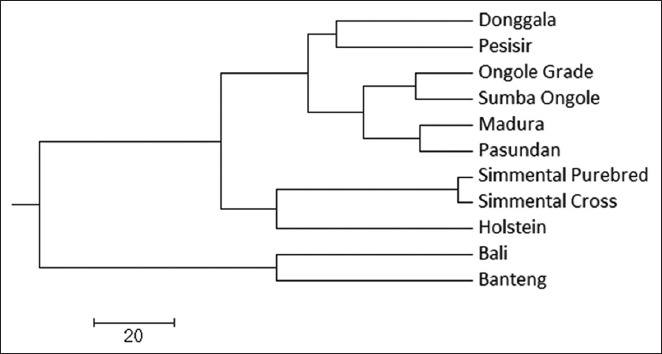
Dendrogram of Donggala cattle and other breeds using the unweighted pair group method with arithmetic mean method based on Nei’s genetic distance [20].

The UPGMA dendrogram indicated that Donggala cattle are genetically closest to Pesisir cattle (Figure 1) [20]. However, despite this genetic proximity, Donggala cattle exhibit larger body size traits resembling Ongole Grade and Sumba Ongole breeds. This suggests a divergence between genetic similarity and phenotypic expression.

### Principal component and admixture analyses

**Figure 2:**
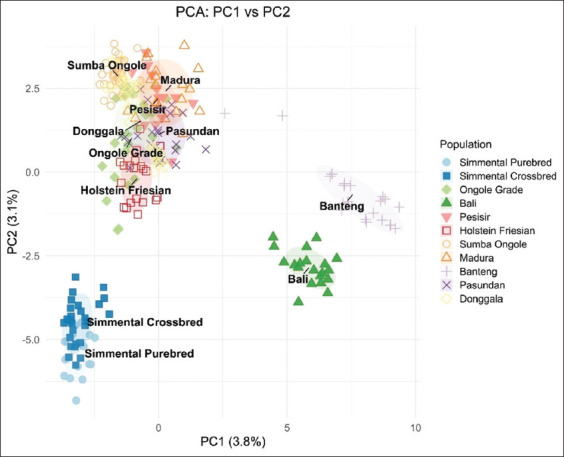
Principle component analysis of 10 microsatellite loci genotypes in the Donggala population and other breeds.

**Figure 3:**
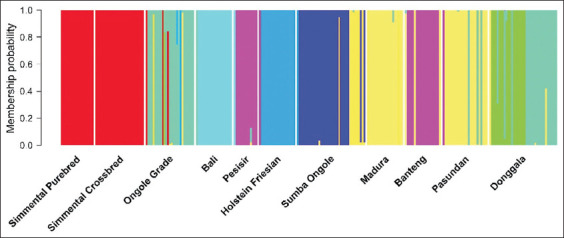
Genetic structures of Indonesian cattle.

PCA results supported the UPGMA findings, clustering Donggala cattle with Ongole Grade, Pesisir, Sumba Ongole, Madura, Pasundan, and Holstein breeds (Figure 2). The unexpected clustering of Holstein with indigenous breeds may reflect PCA limitations in resolving populations with large sample sizes and minimal genetic distances. Admixture analysis produced grouping patterns consistent with the UPGMA dendrogram (Figure 3), reinforcing the observed genetic structure.

### Enhanced resolution with 3D PCA

**Figure 4:**
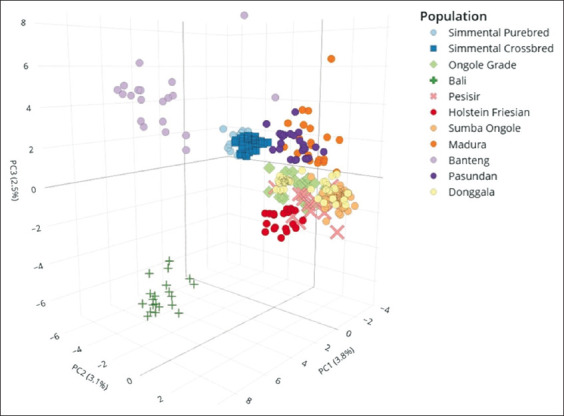
3D principal component analysis of 10 microsatellite loci genotypes in the Donggala population and other breeds.

The 3D PCA provided superior discriminatory power compared to traditional PCA. Donggala Cattle were clearly clustered with *B. indicus* breeds such as Pesisir, Ongole Grade, Sumba Ongole, Madura, and Pasundan, while Holstein cattle formed a distinct cluster (Figure 4). Simmental crossbred cattle were also separated as a unique group. Importantly, the 3D PCA successfully distinguished Banteng from Bali cattle, highlighting its enhanced ability to resolve closely related genetic groups. These results emphasize the effectiveness of 3D PCA for detailed genetic characterization of Donggala and other Indonesian cattle breeds.

## DISCUSSION

### Genetic diversity across microsatellite loci

This study employed 10 microsatellite loci to assess genetic variation in Donggala cattle and compare their genetic structure with other local Indonesian breeds. Although the FAO recommends the use of 30 loci for cattle genetic studies earlier research has shown that analyses with 10 or fewer loci can still provide sufficiently informative insights into population structure and relationships [21–24].

The analysis revealed considerable genetic diversity within Donggala cattle. SPS113 exhibited the highest number of alleles (12), whereas BM1818 had the lowest (3). Allele 264 at BM1818 showed the highest frequency (0.8421), followed by allele 181 at BM1824 (0.421). Such allele dominance suggests possible influences of selective breeding, genetic drift, or founder effects, as reported in other cattle populations [25–30].

### Informative loci and heterozygosity patterns

Among the loci studied, SPS113, ETH225, and TGLA122 emerged as the most informative, supported by high PIC values (0.84, 0.81, and 0.80, respectively) and elevated Ho (ETH225: 0.94, TGLA122: 0.83, SPS113: 0.74). These findings emphasize their value for evaluating genetic variation in Donggala cattle. Such genetic variability is essential for maintaining adaptability, resilience, and evolutionary potential, especially under environmental and disease pressures [31–33].

Interestingly, the He values exceeded observed values across populations, suggesting possible effects of null alleles, inbreeding, assortative mating, the Wahlund effect, or selection against heterozygotes [34]. The genetic relevance of SPS113 and TGLA122 has also been validated in other cattle populations, including Holstein, Turkish native breeds, Portuguese, and Hanwoo cattle, where these loci consistently demonstrated high diversity [35–37].

### Bottleneck and AMOVA insights

Bottleneck analysis under the IAM indicated significant heterozygosity excess in Simmental Crossbred, Bali, Madura, and Pasundan cattle, reflecting recent reductions in effective population size. However, these signals were not evident under the TPM and SMM models, suggesting that the observed deviations may represent historical demographic contractions rather than recent bottlenecks. Donggala cattle, along with several other breeds, showed no significant departures from mutation-drift equilibrium, indicating relatively stable effective population sizes in recent generations.

AMOVA results revealed that variation among individuals (21%) was nearly equivalent to variation among populations (22%), suggesting moderate population structuring. However, the limited number of loci (10 instead of the FAO-recommended 30) and the focus on local breeds may have reduced the resolution of inter-population differentiation. Broader marker panels and wider sampling are needed for more accurate population-level insights.

### Genetic relationships and phenotypic divergence

UPGMA analysis revealed that Donggala cattle are genetically closest to Pesisir cattle, despite their distinct phenotypic traits. While Pesisir cattle are small-sized and native to West Sumatra, Donggala cattle display larger body sizes, resembling Ongole and Sumba Ongole breeds. This divergence between genetic proximity and morphological traits likely reflects environmental influences such as nutrition and management practices, as well as targeted selection [38, 39].

### Population clustering and PCA limitations

PCA grouped Donggala cattle alongside Ongole Grade, Pesisir, Sumba Ongole, Madura, and Pasundan cattle. Interestingly, Holstein cattle also appeared in the same cluster, an unexpected finding likely due to PCA’s limitations in separating populations with minimal genetic distances and large sample sizes [40, 41].

### Enhanced breed differentiation with 3D PCA

The 3D PCA provided greater resolution, distinctly clustering Donggala with other *B. indicus* breeds while separating Holstein into a distinct group. It also successfully differentiated Banteng from Bali cattle, which are often genetically similar. These results confirm the superior discriminatory power of 3D PCA, aligning with a previous study highlighting its effectiveness in complex genetic datasets [42].

### Implications for conservation and breeding

The findings of this study underscore the importance of integrating molecular tools, such as microsatellites, and advanced visualization methods, like 3D PCA, into livestock conservation strategies. The evidence of substantial genetic diversity in Donggala cattle underscores their potential as a genetic reservoir for breeding programs. Incorporating both genetic and phenotypic assessments will be crucial for the formulation of targeted conservation and sustainable improvement strategies for Indonesian indigenous cattle breeds.

## CONCLUSION

This study represents the first microsatellite-based molecular characterization of Donggala cattle, an indigenous *B. indicus* breed from Central Sulawesi, Indonesia. Analysis of 10 FAO-recommended microsatellite loci revealed substantial genetic diversity, with SPS113, ETH225, and TGLA122 emerging as the most informative markers due to their high allelic richness, heterozygosity, and PIC. UPGMA and PCA analyses placed Donggala cattle in close genetic proximity to Pesisir cattle, while 3D PCA provided enhanced resolution by distinctly separating *Bos indicus* from *B. taurus* and clearly differentiating closely related groups such as Banteng and Bali cattle. These results highlight the rich genetic reservoir maintained within Donggala cattle and underscore the value of advanced analytical tools in elucidating population structure.

The findings provide a scientific foundation for the conservation and sustainable management of Donggala cattle, ensuring their long-term adaptability to environmental pressures and disease challenges. The identified loci can serve as genetic markers for future breeding programs, supporting selective improvement strategies at both local and national levels. For farmers, this knowledge offers pathways to strengthen breeding enterprises, improve productivity, and preserve genetic heritage.

Key strengths of this study include the use of well-validated microsatellite markers, comparative analyses with multiple Indonesian breeds, and the integration of advanced multivariate approaches such as 3D PCA, which proved superior in resolving closely related genetic groups. Together, these methods provided robust and high-resolution insights into the genetic diversity and relationships of Donggala cattle.

The study utilized only 10 microsatellite loci, fewer than the FAO-recommended 30, which may limit the resolution of inter-population differentiation. In addition, the sampling was restricted to two sub-districts, which may not fully capture the entire genetic variation of Donggala cattle across Central Sulawesi.

Future studies should expand the marker set to include additional microsatellite loci or SNP-based panels for greater precision, alongside broader geographic sampling to capture regional variability. Integrating genomic data with phenotypic and environmental assessments will strengthen conservation strategies. Moreover, applying genome-wide association studies (GWAS) could link genetic diversity with economically important traits, further supporting breeding and productivity improvement programs.

Overall, this study provides the first molecular evidence of the genetic diversity and structure of Donggala cattle, reinforcing their potential as a valuable genetic resource for Indonesia. The findings not only contribute to the preservation of indigenous cattle breeds but also support evidence-based strategies for their sustainable utilization in conservation and breeding programs. By bridging molecular genetics with practical livestock management, this research sets the stage for enhancing food security, rural livelihoods, and biodiversity conservation.

## AUTHORS’ CONTRIBUTIONS

YD, MT, ADM, and MIM: Conceptualization and methodology. PPA, FS, and SS: Data curation and formal analysis and drafted the manuscript. EMK, MG, TM, NH, and DD: Validation and drafted and reviewed the manuscript. All authors have read and approved the final version of the manuscript.
